# Unmasking the Financialization of Healthcare

**DOI:** 10.1017/amj.2025.10090

**Published:** 2025-12

**Authors:** Jacqueline Fox

**Affiliations:** Joseph F. Rice School of Law, University of South Carolina, Columbia, United States

**Keywords:** healthcare finance, healthcare financing, healthcare reform, health law, health policy

## Abstract

Financialization of healthcare drains our current system of resources it needs to provide care. It occurs when money is siphoned off for private profit through mechanisms such as rent seeking, gamesmanship, and exploitative price setting. This is not an ethically neutral activity, and the people profiting in this way ought to justify why they are entitled to this money, given the foreseeable negative effects what they are doing has on people’s health. This important problem is masked by current accounting methods and healthcare billing methods, which need to be changed to allow for a more transparent assessment of what is really occurring.

## I. Introduction

Our system of accounting for health care costs^1^ is misleading, helping mask significant ethical problems — including resource allocation issues — that ought to be remedied.^2^ Our methods for calculating total spending on health care rely heavily on the cumulative cost of individual patient interactions, represented by diagnostic codes and similar reimbursement methods.^3^ Behind these singular financial transactions meant to track the provision of health care, however, is a complex monetary system that has become focused on health care financialization rather than on “the production of patient and community health.”^4^ Money is being siphoned from the system in multiple ways, creating profit for people who do not provide care but rather harm patient and community health.^5^ These means of appropriation include rent-seeking,^6^ unreasonable price setting,^7^ and inappropriate manipulation of programs meant to subsidize care for those with financial vulnerabilities, which I will refer to as gamesmanship.^8^

According to the definition of financialization used here, the money lost to financialization — including in the three categories of rent seeking, unreasonable price setting, and gamesmanship — is not used to provide health care. Therefore, if we want a true picture of how much we are spending on providing care, rather than a picture of how much our health care financing system costs us, these expenditures must be identified and separated from both the total cost of our system and per capita costs. The exercise here is not merely distinguishing between money spent trying to provide health care and money that genuinely provides it. The distinction is between those who legitimately participate in the health care system and those who siphon off resources that otherwise would go to providing care. The distinction between these two numbers is both theoretically significant and, likely, financially significant.

Furthermore, the monetary cost of harm caused to patients and communities by financialization ought to be separated from the cost of health care; these costs are the costs of financialization, *not* the costs of a system that solely seeks to address illness and injury. For example, unreasonable price setting likely causes patients to delay care in response to overall costs of accessing care, which is associated with worse outcomes and greater expenses.^9^

Section II of this Article focuses on the problems caused by financialization and by masking its costs, showing how little value many of these actors add (even as they cause harm) through a case study of deals involving hospitals, private equity, and a real estate investment trust. Section III discusses the ethical implications of financialization, developing a method for constructing a duty to impose on those who benefit from financialization, one that requires them to justify their profit against the foreseeable harms they may cause to patients and the community. Section IV discusses how this problem is both exacerbated and can be addressed through accounting methodology, particularly given the rhetoric potency accounting results have in discussions about health care spending. Section V is the conclusion.

Identifying and separating non-health care costs from overall costs of the system allows for forthright discussion about the effects of financialization on the resources available for providing health care and about whether this specific type of payment furthers the goals of a health care financing system. The effect is likely big enough to merit the work necessary for this form of accounting to occur. Even with the likely challenges in doing this, a structured recognition of these distinguishable categories of spending ought to make future discussions about resource allocation and funding methods more accurate, rational, and ethically informed.

## II. The Problem of Financialization’s Costs Being Masked

The cost of financialization is ethically important, and, as such, requires justification. Given finite resources, any resource allocation decision is ethically charged, as some may go without necessary care based on these decisions. Our primary rationing tool to deal with the scarce resources available for health care is money, so every act of setting a price and every dollar that goes towards profit will foreseeably cause some harm by limiting some people’s access to necessary care. After all, when a price is set, some people likely will not be able to pay it and will therefore forgo care.^10^ Problematic profit seeking of the type described here is different than, for example, reasonable payment for a person’s labor, such as the direct provision of health care for a patient. The further a payment gets from this type of interaction, both in terms of reasonableness and whether it is directly for the provision of care, the more problematic. It follows that those who profit ought to justify themselves by showing how they have a reasonable claim to these dollars considering the foreseeable likely harm they are causing by their behaviors. They should not have that money without persuasive justification.

It is likely an open question as to whether the difference described here is one of degree or category, but the answer is functionally not important. Given the foreseeable harms caused by all behaviors that relate to cost or distribution, justification is always required. It is straightforward to justify the cost when, again using the example given above, one is paying someone a reasonable wage for their labor, and the work is providing direct care to a patient. It is difficult, if not impossible, to justify taking money from this system when it is profit, derived through gamesmanship or some other form of manipulation.

We have inadvertently legitimized financialization through our accounting methodologies by folding them into overall cost calculations, even as we readily recognize they are problematic.^11^ There is currently no method for reliably calculating these costs. To the contrary, one of the attractive attributes of private equity investing is that it is not subject to the reporting requirements of an investment fund that is subject to SEC disclosures.^12^ People who set prices for drug and medical devices that are subject to intellectual property protection consistently resist efforts to justify their pricing decisions.^13^ Finally, those who manipulate the details of government programs in an effort to maximize profit are unlikely to be open about the money they make doing so.^14^

Currently, to constrain our health care spending and minimize the foreseeable harms of price setting and limited resources, we look downstream for savings, ignoring the predatory financing arrangements, acquisitions, and initial price settings that have already occurred.^15^ This is both problematic and unlikely to yield the desired result of spending our limited health care resources in a manner that provides the most high-quality care for the most people. There may be legitimate room for asking patients, providers, and hospitals to be economical, but it is structurally incorrect to focus solely on this while ignoring the increasing economic pressure caused by financialization. This is particularly true because the profit seeking conduct described in this Article exerts pressure on providers that in turn distorts how care is provided. Focusing solely on downstream savings puts providers under pressure from both profit seekers and cost minimizers, a situation that harms their ability to best care for patients.^16^

For example, private equity investments in health care consistently results in health care costs increasing and quality decreasing, yet we do not account for this distortion when we add up our spending on health care.^17^ We have research showing these problems consistently occur when providers enter arrangements where capital is extracted and debt servicing is imposed — classic components of a private equity deal.^18^ If a provider is pressured to increase patient volume or levels of diagnosis because it has this debt to service, those costs are calculated as merely being the cost of providing patient care, rather than the result of predatory financing mechanisms. This causes us to look more critically at the doctor-patient interaction when seeking savings than at the rent seeker, the ultimate recipient of this extra money now flowing from the practice.

This dynamic can be seen by tracing the various deals and arrangements entered into by Medical Properties Trust (“MPT”),^19^ a Real Estate Investment Trust (“REIT”) that primarily functions as a sale/leaseback company. It purchases the land under struggling hospitals in the United States and then leases that same land back to the hospital that once owned it.^20^ The hospitals make lease payments in return for the capital infusion of the purchase price. The lease payments are like debt because the hospitals must service the payments with income streams, but different than a mortgage because the hospital has given up ownership of the underlying real estate, meaning there is no underlying asset being acquired. The payment made by the REIT in exchange for the sale of the real estate goes to the company that owns the hospital, which can distribute it however it chooses. In a case study discussed below, it was largely used to issue a dividend to the private equity firms that created the entity that owned the struggling hospitals.^21^ These hospital groups routinely enter subsequent new financing arrangements in an effort to refinance unpaid lease payments, resulting in them being sold, entering bankruptcy, or simply being refinanced, with MPT getting a payout from the proceeds (to reduce the debt owed them) even as they also finance the sale by loaning money to the new owner or entity that controls the hospital.^22^

A particularly well-studied set of deals leading to monetary extraction, bankruptcy, and negative impact on patient care occurred when private equity firm Cerberus Capital Management L.P. (“Cerberus”) purchased a set of hospitals in Massachusetts, placing ownership in a newly created entity, the Steward Health care System (“Steward”).^23^ The initial deal took place in 2010, and Steward agreed to be closely monitored by the Massachusetts government for five years.^24^

In 2016, shortly after the monitoring ended, Steward sold the underlying real estate owned by the hospitals to MPT for $1.2 billion. Cerberus immediately used $484 million of that for a dividend to shareholders, even as the financially stressed hospitals were now responsible for new lease payments.^25^ In 2020, after the hospital group rapidly expanded from eleven to thirty hospitals, Cerberus was bought out by a physician group who funded the purchase with a $335 million loan from MPT.^26^ There were many other subsequent complex financial arrangements where hundreds of millions of dollars in value were pulled out by various investment companies, saddling the hospitals with increasing and complex debt obligations.^27^ On May 6, 2024, Steward filed for bankruptcy protection “reporting over $9 billion in liabilities,^28^ $6.6 billion of its reported liabilities . . . [were] long-term lease liabilities owed to Medical Properties Trust.”^29^

Much of that $9 billion was not used for providing health care. Looking at congressional testimony, witnesses gave numerous reports of quality plummeting after Steward purchased the hospitals, especially after payment obligations increased due to the financing mechanisms being used.^30^ Detailed testimony reported many preventable patient deaths due to lack of providers or resources, as well as unpaid health care providers and unpaid medical supply companies.^31^ The people doing the actual work were not being paid,^32^ the people providing actual supplies were not being paid,^33^ and patients were receiving terrible or no care.^34^ It reached the point in some Steward hospitals that the Centers for Medicare & Medicaid Services ("CMS") threatened to refuse to pay for Medicare patients to be treated there because the hospitals presented a danger to Medicare beneficiaries.^35^

It is rare to have the specific numbers that we have in this case study; private equity firms (and similar investment companies) have limited reporting obligations, and the various financing arrangements are complex and opaque.^36^ However, even without an organized and coherent methodology for tracking this money, studies show that the Cerberus case is not an outlier, but is, rather, an example of the problems private equity is causing to the health care financing system.^37^

While many states and the federal government are considering or enacting legislation to constrain financialization in health care,^38^ legislative action is likely not sufficient absent a clear ethical framework that convincingly shifts the burden of justification onto those who profit. Those who profit from financialization voluntarily enter the health care market with a business goal of making money in a way that does not provide care.^39^ Absent a persuasive justification showing that benefits to patients offset the foreseeable harms they cause, taking money from this health care system in this way is ethically wrongful and ought not occur. Our system of accounting methodologies should identify these profiteers and require them to present these justifications, rather than inadvertently assist them in masking financialization’s harmful impact. It ought to also clearly track the profit and subsequent strain on resources.

## III. Ethical Duties Accompanying Financialization in Health care

When a company seeks investment income or seeks to profit from manufacturing goods and chooses to do this work in the health care field, it has decided to voluntarily enter a field where its business decisions have foreseeable effects on people who are sick and injured and in need of care, all of whom will eventually die. This frailty is an inescapable part of the human condition, but it plays a particularly pointed role in the background conditions accompanying any work that relates to health care, where human frailty is the underlying reason for the field. The importance of how patients are treated is evidenced by the stringent professional ethics codes^40^ that recognize the vulnerability of patients and the ethical ramifications of care provider behavior.

A profit seeker’s choice to participate in this field should be accompanied by ethical duties because of the potential ramifications of pricing and profit-seeking behavior in this field. These ethical duties ought to both prohibit certain types of behavior and require persuasive ethical justifications for others.

There are two specific ethical obligations relevant and discussed here. First, the high stakes of people’s interactions with the health care system give rise to the potential for tremendous exploitation, as people will likely pay any price for medical care they desperately need.^41^ Participants in the healthcare system are ethically required to not behave in an exploitative manner, a duty consistent with the overarching ethical requirement that all people refrain from exploiting others.^42^ In health care, where dramatic vulnerabilities are more readily exploitable, it is important to explicitly consider the real possibility of causing harm and minimize this risk by designing an ethical analysis that leads to understandable duties for appropriate behavior.^43^ Second, both pricing decisions and other forms of profit seeking have foreseeable negative downstream effects because they reduce available resources for everyone in the system; these decisions ought to be made with the understanding that the profit sought must ultimately be defended within ethical parameters.^44^

Not considering these issues *is* an ethical choice. Structuring a system where people have the power to make unethical decisions is also an ethical choice. This article identifies these ethical moments and calls for a specific structure for approaching them.

One can create a coherent, ethical, and internally consistent approach to profit seeking in the United States health care financing environment, but it requires both a reordering of what counts as health care spending and a reordering of the ethical obligations we place on those who profit in any way from the system. This is why accounting methodologies, and the law underpinning them, are relevant here.

We have done much work to achieve a modern understanding of social justice theory and social science research. Consistently, both research and theory show us the limits of competition and free markets to correct health care resource misallocations in our financing system,^45^ yet we have not centered this understanding in our relationships with those holding primarily financial roles in the health care system. We inadvertently privilege the financial wellbeing of rent seekers and price setters over the patients the system is meant to help; we do so by not tracking money spent on profit-seeking behavior and asking profiteers to get paid less money — even as we ask *others* in the system to do more with less.^46^ This is ethically insupportable.

Much of the money that flows through the health care system goes towards financialization schemes such as rent seeking, prices that exceed a reasonable value of the good being paid for, and intentional manipulation of programs meant to subsidize care for vulnerable populations. Even though this is technically money spent in the United States health care system, it is not money spent on health care, and we should be more careful about this distinction.

As we adjust how we account for the money going through this system to clearly identify these categories, we ought also to invert the hierarchy of where we commonly look for savings, first focusing on those who provide no or little direct care rather than on patients or doctors. Asking patients and doctors to save money is stressful and potentially harmful.^47^ Reducing or eliminating profit streams that are not adding anything meaningful to the health care system is a more attractive target of cost savings. Given the foreseeable negative downstream effects of financialization, the ethical justification for this flip is even more persuasive.

One who intentionally enters the health care marketplace because one seeks to profit from it while providing no meaningful care to patients, ought to have a heavy burden of justifying why they are entitled to any money from that system. If the reason we put money into the health care system is to provide health care, money that does not do so is being improperly spent. It is important to remember that one can seek profit in any industry, and the health care system does not owe a profit seeker any sort of opportunity by default.

One can readily imagine a sufficient justification for many common financial transactions. Renting an office building, loaning money for a new wing to a hospital, etc., could be done in a manner that is not inherently problematic, and could be justified as helping providers provide care. The justification is that the transaction or the actor provides a good that, in balance, is worth the foreseeable negative effects on access and care that come from the pricing and/or profit seeking behavior. Having a burden of justification is not a subterfuge for preventing all transactions, it is meant to function as a tool for preventing ethically impermissible transactions.

The typical private equity firm investing within the health care field requires this analysis. Though often promoted as beneficial for health care because of developing efficiencies while freeing up capital for the acquired health care provider,^48^ the mere fact that these firms commonly justify their role in health care by asserting these claims implies an inchoate understanding of some ethical burden, or at least a recognition of the problematic optics of profiting from health care without even a pretense of improving outcomes.

Imposing an ethical duty on those who seek to profit from the financialization of health care allows one to rebut private equity’s justifications by asking for an explanation as to why these ‘efficiencies’ are sufficient as a justification for the foreseeable harms caused by the financial transaction. One can also ask for evidence that any such efficiency (the first justification) is achieved, which seems hard for these firms to argue persuasively. As has been cited throughout this article,^49^ numerous persuasive studies show poorer outcomes, increased provider burnout, and increased patient costs after private equity purchases a health care institution.^50^

The second justification, the release of capital, is perhaps more interesting. The use of the capital that is released through the financing mechanisms of purchase or sale leasebacks, etc. is not constrained, so it does not have to be used for anything related to the mission of the provider or hospital.^51^ As in the case study about Steward described above,^52^ the money often funds a significant dividend for shareholders of the private equity firm that designed the financing mechanism that collected the money. The practice or hospital is left with the debt but often little of the money the debt is evidence of. It is shocking that such an intentional wasting of health care resources has been allowed. Requiring a justification related only to the provision of health care would make such a transaction insupportable. It could also function as a reason for prohibiting taking the value of a health care institution’s underlying assets away from it to profit those disconnected from that institution.

Unreasonable price setting also comes with foreseeable harms. There are people who cannot pay for care at that price and so go without. When the price is paid, the unreasonable price improperly drains resources from a system that has finite resources. This later harm is experienced by all persons who must function within a system that has fewer resources available to provide all types of care.

Those who set prices are looked at differently in this ethical framing than how they are commonly viewed, with far less privilege and a strong duty of justification for every choice they make.^53^ The health care system is intimately concerned with people who are passing through risky, painful, and stressful stages of their life. Its pricing mechanisms should not allow the ultimate threat of untreated illness or injury to weigh into calculations. The goal ought to be creating a system where prices reflect fair reasonable payment for labor and produce fair returns for taking financial risks when pursuing innovation, not one with the potential of extortion in the face of threats to one’s very life.

Calculating prices for health care based on what would happen if care were not provided is akin to calculating payment for a barrel of water in a drought. You must have the water to live, so of course *any* trade of *any* value is rational. But it is better to build a system for delivering water in an affordable and reliable manner.^54^ Calculating a price for that water in a manner that internalizes the exploitative power one has over people who would otherwise die of thirst is absurd. If we assume that exploitation is unethical, a properly functioning water distribution system ought to be constructed to fairly pay for the labor and intellectual prowess that is involved in providing water; the primary organizational goal would be rational and efficient water distribution, as well as avoiding the potential for exploitation.^55^ Seizing control of the water supply and threatening to kill someone by not allowing them access is, at best, exploitation and, at worst, murder.^56^

Nicholas Bagley discusses something similar looking at health care from a public utility perspective and describing “an affirmative obligation … to be reasonable in dealing with the public[,]” particularly when actors “serve[] an important human need and have the market power to exploit consumers … .”^57^

We do utilize methods for incorporating rational and ethical concerns into pricing, primarily through cost effectiveness analysis. Scholars have done good work examining this process and theorizing methods within it to achieve better results. For example, Govind Persod, in *Pricing Drugs Fairly*, devises a method for correcting overpricing, recognizing, and pushing back against, market exclusivity problems and stark dependance that patients have on the drugs they need.^58^ His theory, in relevant part here, is that pricing ought to be fair in relation to the social value of a drug.^59^

However, comparative effectiveness analysis for pricing purposes brings these issues into the system at the wrong place. Conceptions of value and benefit ought to be implemented at a structural level as well as at a specific comparative pricing moment, where we do such an analysis to push back at the prices drug companies are seeking.

Comparative effectiveness asks us to consider the cost of an illness and the savings that a treatment offers us. We consider a rational price for the treatment in light of how much money, suffering, and life years it potentially saves us. We ought not be framing the question this way, as doing so internalizes our acquiescence to negotiating with highway robbers. Embedded into our current framing of the best price is the possibility of going without treatment if a price cannot be agreed upon.

Even in a comparative effectiveness discussion where a powerful purchaser is negotiating pricing, the manufacturer needs to be cajoled into an agreement even as it holds people’s very lives over their heads. The current system gives companies the power to walk away, with potentially devastating effects on health care outcomes, because barriers to entry make it implausible that another entity can take their place.^60^

We are beginning to formulate methods for demanding a more rational stance when negotiating prices.^61^ The overarching ethical goal should be fair and reasonable pricing, stripped of the threat of preventable death and suffering. Even if this is not plausible given our current system of market exclusivity and steep barriers to entry, we should certainly be acknowledging the threat for what it is and publicly air its wrongfulness.

All actors in health care financing are responsible for the ethical ramifications of how they behave, morally culpable for causing harm when they cause harm. We should require these actors to defend the harms they cause within a framework that sets expectations of ethically proper behavior and transparently discusses what is being balanced when prices are set.

Companies working and setting prices within this system come to the industry by choice, voluntarily entering a system where their decisions have profound ethical implications. Expecting ethical justifications for the effects of these decisions is clearly not radical. It is more extreme to accept the harms to people’s lives and health caused by extreme pricing as somehow natural (rather than constructed) and seek solely to minimize those harms *after* the price has been set.

Another way to describe this flaw comes from examining the concept of value as it applies to health care and then looking carefully at how financialization fits within it. As Michael Porter wrote, “[i]n health care, value is defined as patient health outcomes achieved relative to the costs of care. It is value for the *patient* that is the central goal, not value for other actors per se. In a well-functioning health care system, the creation of value for patients will determine rewards for all system actors.”^62^

If this definition holds, value-based purchasing is meant to enhance a patient’s outcome relative to the cost of care.^63^ Furthermore, if someone is currently benefitting from the health care financing system but does not create this exact type of value, they ought not have any rewards from this system. This logically implies that, as a necessary condition of participating in the system, all participants must be able to explain how they increase value for patients.

I would argue that it goes further. Given the ethically fraught implications of a health care system with scarce resources, participants have a normative duty to clearly show they increase value in meaningful ways. Value ought to be present before someone can profit and the degree of value ought to be tightly related to the amount of profit.

Using the lens of value allows us to focus on the concepts of over-valuing and undervaluing. Within the definition of value given above, a participant in the system is over-valued if it is rewarded in excess of the value it brings to patients. While achieving numerical clarity to answer this may be difficult, the logical claim is tight. The creation of value determines the reward.^64^ So, it is possible to infer that first, some value is required for there to be any reward, and second, the reward is calculated proportionally to the value.

We currently pursue value in interactions between and among hospitals, providers, and patients, while not directly focusing on these other large players in the system as described in this Article — that ought to change. This value framing identifies the requirement that any participant in the health care field must bring value to patients to profit. The definition of value, and the limits on it, however, must be constrained, likely with some form of the public utility argument given above, so that it does not become another opportunity for exploitation of the life-or-death nature of health care. Preventing death and suffering *is* extraordinarily valuable but is also the *purpose* of the system.

Finally, given the importance we place on health care spending in any given year, the method for getting that number matters. If that methodology masks the impact of the financialization of the health care system, it distorts our decision making about health care financing reform. We consistently burden patients and providers by asking them to do more with less without making sufficient efforts to minimize that burden by protecting the assets that are potentially available.

By folding the costs of financialization into the overall costs and per capita costs of the health care financing system, we are building that profit on patient’s and provider’s backs, calling on them to justify high costs and shoulder a greater share of this burden. When you look to the patient and to the person buying insurance for savings, it implies they need to pay more, that they are somehow getting more than they ought to, and so they are appropriately the first place to look for constraining overall spending. When we ask providers to be more efficient, to see more patients and still maintain quality, we are saying that they are currently doing something wrong, taking more than their fair share of resources and time.

The folk singer Jesse Welles has a song that begins “If you worked a little harder, then you’d have a lot more. So, the blame and the shame’s on you for being so damn poor.”^65^ In this song, he describes our perception of poverty as caused by failures perpetrated by the person with limited resources.^66^ This view of poverty is inaccurate; the way our society is constructed makes it difficult for people to ‘work[] a little harder’ so they aren’t so poor.^67^

Welles’ lyrics are directed at poverty and how we blame people who are living in poverty for what society does to them. This same dynamic occurs in health care financing, where we seek savings from patients and providers even as we realize many other people profit in ways described in this Article. Focusing on these people to fix cost problems implies the system would not be this expensive if patients and doctors did not act the way they do. For example, there have been repeated claims that patients are likely to seek care they do not need when the cost is low.^68^ We ask patients to carry more of the risk of insurance by having extremely large deductibles under the theory that having ‘skin in the game’ will foster better choices by patients.^69^ This rests on a strange construct that somehow people *without* medical training are the ones who ought to determine when care is necessary and need powerful financial incentives to constrain them.^70^

Unsurprisingly, even as we have public health campaigns seeking to educate people about the symptoms of emergent conditions such as heart attacks and strokes,^71^ financial incentives to delay care such as high deductibles have a powerful, negative impact on patient’s rates of seeking care.^72^ Given the downsides of these efforts to save money at the patient-provider level, we should also look to those who profit from financialization for savings.

## IV. Accounting Methodologies and Rhetoric

Ensuring that we maintain focus on identifying and minimizing spending that does not provide health care serves purposes beyond ethics, justice, or fairness: it increases accounting accuracy, allowing policy choices to be made from a more informed perspective.^73^ By failing to do this, our current system distorts internal discussion and international policy making. We recognize that, according to our accounting principles, we spend more on health care, in total, than other countries do, we spend much more per capita than other countries do, and our returns on this investment are persistently poor.^74^ We constantly confront evidence of poor outcomes through multiple lenses.

Internationally, we have measurably worse outcomes across the board, particularly when compared with countries of similar financial robustness.^75^ These problematic outcome statistics include overall life expectancy,^76^ maternal fetal morbidity and mortality rates,^77^ and mortality from treatable causes.^78^ Within our country, we also see wide and indefensible variables in outcomes and other measurements of quality, where the socioeconomic identity,^79^ race,^80^ gender,^81^ wealth,^82^ education level,^83^ Body Mass Index,^84^ and a myriad of other seemingly irrelevant qualities of a patient coupled with their geographic location^85^ often serves as a more reliable predictor of outcomes than the immediate health problem they are facing.^86^ These outcome differences often persist even though the per capita spending (as we currently calculate it) for these patients may be equal to or higher than what is spent on someone likely to have better outcomes because of having more privileged qualities.^87^

The problem seems exceptionally puzzling and complex when the number of how much is spent is accepted at face value; if money buys access to health care, and we are paying that money, why do we fail to buy good outcomes? If we throw money towards treating illnesses in vulnerable populations, why are we failing to buy good outcomes there?

Perhaps we need to consider the possibility that we may not spend that much money on health care, we may not actually spend more than every other country per capita on providing care, and in fact spend far too little on providing care across our system — particularly in areas where the outcomes are poor — and this lack of spending contributes to our poorer outcomes.

To investigate whether we may be spending too little would require us to consider that our methods of calculating health care spending are incorrect when considered in light of how the health care financing system in the United States is constructed. The financialization of health care coupled with our current accounting methods may be distorting discussions about resource allocation. The political debate would likely be very different were one to acknowledge that people are going without necessary, timely care so a private equity firm can declare a dividend, *not* because health care resources are too scarce to go around.

It is not unprecedented to raise a claim that an accounting process is incorrect and may be doing harm, though these claims generally arise within the profession of accounting, and not as often outside of that specific field.^88^ Separate from the concerns raised in this Article about the propriety of rent seeking, etc., people outside of accounting tend to accept that the numbers presented as a result of compliance with accounting methodologies tell us something true that they are not capable of doing, as though it is a simple question of counting countable objects. This distortion occurs, in part, because of the powerful rhetoric that accounting contains.

If one was simply counting a barrel of sixty-five apples in a careful and reliable way, one would know something specific and tangible: there are sixty-five apples, so sixty-five people could each get an apple. Health care accounting is presented as if it were simply counting apples, but it is not. Accounting standards (the terminology used to describe agreed-upon accounting methodology) are negotiated and subject to routine change, with the understanding that the changes will alter the final numbers.^89^ The discussions in these negotiations often contain explicit recognition of the ethical and sociological values that are embedded in the adopted rules.^90^ Furthermore, within accounting, the outsized power of the rhetorical content of these numbers is widely recognized.^91^

The communications of accountants to the non-accounting world — such as our national yearly health care spending^92^ — have both potent rhetorical content and risk of doing harm. The rhetorical weight leaves numbers vulnerable to misunderstanding and casual usage that is not necessarily tightly connected to what the numbers reflect. Within academic accounting, there is common and open discussion about the potency of this rhetorical communication and its flaws.^93^ For purposes here, it is enough to recognize that the numbers used in accounting have a rhetorical content which implies they express a true statement of fact, whereas in reality these numbers are evidence of significant compromise and value debates and are ultimately changeable.^94^ Because accounting methodologies reflect these choices, they are instruments of communication about values that are imbedded in those choices, far more complex than simply counting apples.

The process of adding up how much we spend on health care is a method of calculating economic statistics used to make policy. These numbers are used, for example, to compare us to other countries,^95^ to tell us how much we value health care,^96^ and to shame us with how wasteful we are.^97^ They are rhetorically weighty.

While gross and per capita health care numbers have not been subject to close criticism regarding their capacity to cause harm, similar forms of statistics have been elegantly analyzed as political artifacts^98^ and as error laden constructs with the potential to cause harm, constructs that often serve to mask outright dishonesty and other forms of subterfuge by those seeking profit while manipulating policy discussions.^99^

An example of the faultiness of these statistics, and of how they can be used to mask problems within a society, particularly through comparison with other countries, is employment statistics.^100^ Daniel Mügge makes a persuasive argument that the methods for collecting employment statistics spring from white men working for factories in developed countries.^101^ The numbers have no mechanism for reflecting exploitation ranging from lack of bargaining power to outright enslavement, though it is obvious these drastically alter employment statistics in terms of hours worked, productivity, the cost of labor, etc., as well as raising deeply problematic ethical issues that are masked by the anodyne numbers employment statistics provide.^102^

If we look at the governmental communications about how much is spent on health care, you see language that communicates high levels of confidence in the numbers being given, particularly in the United States.^103^ Confidence in the value of the numbers makes sense, on one hand, because it is premised on compliance with accepted methodologies used for determining these numbers. It is inappropriate, on the other hand, if one acknowledges the known limitations of what these numbers reflect.

In the United States, the Center for Disease Control (“CDC”) web page offers this statement: “[the United States] health care spending grew 7.5 percent in 2023, reaching $4.9 trillion or $14,570 per person. As a share of the nation’s Gross Domestic Product, health spending accounted for 17.6 percent.”^104^ The number $14,570 is precise and crisp. The paragraph above that number acknowledges that the National Health Expenditure Accounts (“NHEAs”) are estimates but gives no further context or discussion of the effect of it being an estimate.^105^ Instead, the next sentence claims that NHEA measures these expenses and collects data to do so.^106^ There is no further discussion of limitations on the validity of the numbers.^107^

Shifting from the problems and risks inherent in accounting, consider how the accounting processes for calculating health care expenditures mask financialization. Consider single payer health insurance for a moment, particularly how we calculate its prospective cost in the United States, and the errors these calculations contain due to the problems described in this article. In a 2020 article, Cai and Ostrer et al. did a systematic review of several economic analyses of these programs and found that the majority of the studies showed single payer plans would generate cost savings.^108^ These studies all assumed prices would go down, due to government bargaining power constraining prices, and utilization would go up, due to people accessing necessary care because cost impediments would be removed.^109^ The Congressional Budget Office has also done similar scaling of these proposals, finding that rates would go down, utilization would increase, and resources would be strained, concluding that the overall costs of health care would go down while government spending would increase.^110^

The general tussle between optimistic and pessimistic assessments of the overall cost of such a system seems to come from differing perspectives of the impact of getting access to care for those who currently do not have it, a somewhat ghoulish acknowledgement of the limitations of our current system.

The boundaries drawn on what is counted in the system include doctors, hospitals, and drug costs, as well as anticipated patient needs, but do not include any financing mechanisms or the known effect these mechanisms have on provider behavior.^111^ Given how much data we have showing many of these mechanisms cause increased utilization, upcoding, lower quality, bankruptcies, employee dissatisfaction, etc.,^112^ even without tracking the net loss of money from a strained system, it is problematic to not account for how constraining these problematic investments could change the net costs, and to not address whether any specific plan has mechanisms to do so.

Notably, absent specific mechanisms, it is likely incorrect to assume a single payer system would constrain these participants or their profit, which could substantially change current financing calculations. The UK has national health insurance and increasing value extraction by private equity, so these are not mutually exclusive.^113^ A May 2024 article collecting studies of the effect of private equity on quality and access shows similar effects of private equity investment to what has occurred in the United States.^114^ Allowing private companies to provide care to the National Health Service (“NHS”) patients has been an undercurrent project of Tory politicians for some time.^115^ Tory governments underfunded NHS, which, combined with COVID, led to increased wait lists, which increased public acceptance of private firms being used, even by Labor governments, to reduce the waits.^116^ Private equity, not surprisingly, has been lobbying for this change and is a primary financer of the private institutions providing the care.^117^ Labor’s shadow health secretary Wes Streeting acknowledged that if their party were in the majority, they would likely use these companies to drop the wait lists and that, when it occurred, he would “be pretty furious at the costs involved, because it shouldn’t be the case that because Tory governments run down the NHS, we have to spend more taxpayers money than would be necessary in the private sector because we haven’t sorted out the public sector.”^118^

We see in this story from the UK that failing to account for financialization leads to significant flaws in the premises underlying the debate about altering the health care financing mechanisms in the United States. It is difficult to have accurate policy debates and consider amending or revising financing mechanisms properly without grasping the extent to which the background pressures of financialization distort our current system and create unknown risks for distorting any new system. Because there is so much money to be made, these profiteers will find their way into any system and therefore must be looked at directly and planned for. Accounting, though it currently helps mask these profiteers, could change to help us achieve this improvement.

It is challenging, certainly, to tease out how this ought to be accounted for, but that does not change the definitional problem. Doctors being paid for providing care is a different category of spending than investors being paid for structuring a deal. Accounting, in turn, ought to be sophisticated enough to properly account for this categorical difference. Otherwise, any number that purports to show how much the United States spends on health care per year, or how much it spends per capita, is misleading.

If one is persuaded by the argument presented here, that some types of spending within the health care system are not being spent on health care, per se, but are being extracted through the financialization of health care, this number ought to be corrected. It may help to consider this as a formula, one which shows the algebraic relationship between financialization of health care and the actual cost of health care.

This formula shows how to conceptually cordon off the costs of financialization, as distinct from the costs of providing health care. N is the amount we currently claim to spend on our health care system (and N/# of people in the United States is the per capita amount).^119^

Having an algebraic method of expressing how financialization distorts N is necessary for two overlapping reasons. First, N is incorrectly portrayed as a statement of how much money the United States spends on health care. Instead, N tells us how much money flows through the system, whether it is spent on health care, methods of financialization, or other costs.^120^ Second, given the potent rhetorical communication that N represents in policy and public debates about health care financing, showing the logical relationship between financialization of health care and the remaining true cost of health care allows for a more informed discussion about who ought to be receiving the money that flows through the health care system.

This formula can be approached in two manners. In both, N is what we spend on our health care financing system and HS is what we truly spend on health care. The first is as a discount rate, where one reduces N by the percentage of all cost that goes to financialization, the F rate, to get HS. For example, if data showed that the effect of F was to increase costs by twenty percent, we would multiply N by .83 (the eighty percent of costs that go towards health care) to find HS. The second is simple arithmetic, N-F=HS, where F is the money going towards financialization.

F in both scenarios is the sum of R (rent seeking costs), P (unreasonable price setting costs), and G (money lost to gamesmanship), though these are not finite categories and likely often overlap.

Much of the recent data about the problems with financialization have shown proportional increases, for example, that when private equity buys a practice, billing increases by a certain percentage.^121^ It may be, then, that N is reduced by a percentage, rather than by subtracting a specific number. So, if R, P and G are percentage increase in costs due to private equity, we would calculate a multiplier that would give us a discount rate. However, were accounting and reporting rules changed so that there were more accurate numbers available as to what was truly being spent in these areas, proportionate discounts would not be necessary.

For those familiar with torts law, the idea of using a formula to show the relationship between concepts when seeking to find the scope of a duty is familiar, given Learned Hands’ *Caroll Towing* formula, where, with regards to the duty of care for a moored boat, he found

the owner’s duty, as in other similar situations, to provide against resulting injuries is a function of three variables: (1) The probability that she will break away; (2) the gravity of the resulting injury, if she does; [and] (3) the burden of adequate precautions. Possibly it serves to bring this notion into relief to state it in algebraic terms: if the probability be called P; the injury, L; and the burden, B; liability depends upon whether B is less than L multiplied by P: i.e., whether B > PL.^122^

The Learned Hand formula is used to determine what is reasonable by showing the relationship between individual or societal burdens and the probability and gravity of foreseeable harms to others.^123^

A central issue in *Caroll Towing* was the likelihood and severity of damages.^124^ Here, if the goal is to determine the true cost of financialization within the health care system, and that this is done with an eye towards formulating a duty such that those who profit from financialization must justify that profit in light of foreseeable harms it causes, the formula has to change to look outside the immediate calculations already described and include these damages.

Presuming, as argued above, that financialization causes foreseeable harms and increases the risk of harms occurring, that cost ought to be included. To accurately describe N, as it is currently calculated, then, it would be N=HS+F+C, where C is the societal cost of the harms caused by financialization that are in addition to whatever money leaves the health care system due to specific incidences of financialization. A simple example of this would be the additional cost of treating an advanced disease that was not treated early due to excess price setting for an earlier stage treatment. To calculate what we spend on health care, excluding costs spent repairing the harms caused by financialization, then, we get N – (F+C) = HS.

Considering the cost of health care without any excess spending generated by the foreseeable harms caused by financialization allows us to understand what health care costs would be without financialization. The formula also allows us to fully appreciate the harms that financialization causes and increases the burden of justification on those who benefit from this type of behavior since the full cost of it is now placed on their side of the ledger.

## V. Conclusion

There are flaws in health care accounting, and some of those flaws are currently helping to mask the financialization of the system. By not counting money that flows to those who are profiting from financialization, they are absorbed into the overall costs, and the impact is disseminated across large numbers of individual patient bills, rather than being calculated as a distinct and likely unnecessary burden on the scarce resources we have for health care spending. By not identifying the excess costs generated by the harms caused by financialization, we further mask how damaging these behaviors are.

Those who benefit from this profit ought to be identified and their profit calculated. They also ought to justify their claims for this money, given the foreseeable harm that is caused by extracting money from this system. People go without necessary care or suffer significant financial struggles and at least some of that suffering is for the purpose of supplying other people with a profit, even as they give no health care in return. Accounting has been coopted so that it functions as a shield for those who extract profit, rather than as a tool for holding all participants properly accountable.

The law is a potential tool for correcting these problems. Ethical rules that constrain providers is another possible tool. Any changes that are proposed would be more effective and the arguments in favor of them more persuasive if we adopt a clear ethical statement of duty regarding profit in health care and alter accounting methods so that we have a clear picture of who profits and of how much they make.

## Footnotes

1 The United States uses the National Health Expenditure Accounts (NHEA) system of accounting and also uses the data collected for that to report health care expenditures for international comparative purposes using the System of Health Accounts 2011, similar to most countries. *See* Ctrs. for Medicare & Medicaid Servs., *National Health Expenditure Accounts (NHEA)*, Ctrs. for Disease Control: Nat’l Ctr. for Health Stats. (June 9, 2025), https://www.cdc.gov/nchs/hus/sources-definitions/nhea.htm [https://perma.cc/2LEX-BAQA]; Michael Mueller & David Morgan, *New Insights Into Health Financing: First Results of the International Data Collection Under the System of Health Accounts 2011 Framework*, 121 Health Pol’y 764, 765 (2017).

2 The United States has two systems for calculating our expenditures. *See supra* note 1.

3
*See* Ctrs. for Medicare & Medicaid Servs., National Health Expenditure Accounts: Methodology Paper, 2023 4, 6 (2023) (detailing calculations for cost of patient interactions). The federal government publishes useful highlights to the NHEA and, in this report, breaks down the categories of expenditures. *See* Ctrs. for Medicare & Medicaid Servs., National Health Expenditures 2023 Highlights 1, 2 (2023), https://www.cms.gov/files/document/highlights.pdf [https://perma.cc/7R9G-M5UR] (hereinafter Health Expenditures Highlights).

4 The phrase “health care financialization” is commonly used to refer to companies seeking profit through financial strategies rather than through creating value in the marketplace. *See* Alexandra Allen, *Financialization in Health Care: History, Current Trends, and Impacts on Patients*, Healthcare Value Hub, no. 47, Nov. 2024, at 1, 1, https://healthcarevaluehub.org/resource/2024/financialization-in-health-care-history-current-trends-and-impacts-on-patients/ [https://perma.cc/DF7Q-EURW] (discussing trends of financialization in health care); Erin C. Fuse Brown & Mark A. Hall, *Private Equity and the Corporatization of Health Care*, 76 Stan. L. Rev. 527, 532-33 (2024) (describing health care financialization as increased pressure “to put profits over patients” that may result in decreases in quality of care). As described by Erin Fuse Brown, “health care financialization refers to the shift in the primary objective of health care institutions from the production of patient and community health to the extractive production of wealth for equity owners and management.” Erin Fuse Brown, Professor, Brown Univ., Presentation at ASLME HLP25 (June 5, 2025).

5
*See generally* Joseph Heath, Morality, Competition, and the Firm: The Market Failures Approach to Business Ethics (2014) (discussing trends of market failures). Joseph Heath has a virtue ethics-type argument about business ethics that works well for describing what is problematic in situations where for profit actors seek profit within health care while knowing health outcomes will worsen as a result of their behavior: “[I]n cases where the explicit rules governing the competition are insufficient to secure the class of favored outcomes, economic actors should respect the spirit of these rules and refrain from pursuing strategies that run contrary to the point of the competition.” *Id.* at 5.

6
*See* Brown & Hall, *supra* note 4, at 531, 536-37, 594. Another term for this is profit extraction, as used by Professor Brown in her scholarship in this area. *See* Brown, *supra* note 4. The term rent seeking is used here to describe an entity removing money from a system without creating value. *See* Jathan Sadowski, *Lords of the Platform: Rentier Capitalism and the Platform Economy*, *in* Platform Labour and Global Logistics: A Research Companion 28, 28-38 (Immanuel Ness ed., 2023) (describing limitations placed by rent extraction on those entering the market); CFI Team, *Rent-seeking*, Corp. Fin. Inst., https://corporatefinanceinstitute.com/resources/economics/rent-seeking/ [https://perma.cc/BPA9-DBE3] (last visited Oct. 27, 2025) (defining “rent-seeking” as occurring “[w]hen an individual or an entity seeks to increase their own wealth without benefiting society” (alteration in original)). It is commonly used this way in economic and policy literature, and it is critical, referring to specific types of behavior. For example, see generally Sadowski, *supra.* One could take a position that any form of capital extraction that is not illegal is permissible and, being permissible, requires a neutral or passive descriptive term for the behavior. This Article does not do this, instead defining it in such a manner so that one who creates value within the health care system will only be called on to justify excess profit and one who does not create this value is subject to criticism for extracting scarce resources for private purposes. An example of rent seeking, as discussed further at page 10, is private equity firms taking over a hospital and selling the real estate in a sale-leaseback, then distributing the money from that sale to owners of the private equity firm rather than using it for the hospital, though the hospital now has to pay the lease payments for the land it once owned outright. *See infra* text accompanying notes 19-22.

7 Matthew Speer et al., *Excess Medical Care Spending: The Categories, Magnitude, and Opportunity Costs of Wasteful Spending in the United States*, 110 Am. J. Pub. Health 1743, 1743, 1745-46 (2020). Inaccurate and excessive price setting is categorized as a form of waste. *Id.* I carry it somewhat further than they do, though I think it fits, conceptually, within their framework. Excess pricing is unreasonable and wasteful, but I believe it should not be measured entirely with reference to comparative effectiveness and similar concepts, that there is an ethical requirement for limiting cost that precedes proof of effectiveness. This is discussed more fully below. *See* discussion *infra* Section II.

8 This term comes from the work of Professor Brown, who refers to this behavior as “gaming the system.” Brown, *supra* note 4; A well-known example of the risk of gamesmanship would be the exploitation and distortion of the federal 340B drug discount program. For a discussion of the various entities exploiting the program, see Joseph Walker, *Employers Get Big Drug Discounts Through Program for Hospitals that Serve Poor Patients*, Wall St. J. (Mar. 15, 2025, at 09:08 ET), https://www.wsj.com/health/healthcare/prescription-drug-prices-340b-pharmacy-benefit-plans-6834cc08 [https://perma.cc/SU9H-9KKL].

9
*See* Chidimma D. Azubuike & Oluwatobi A. Alawode, *Delayed Healthcare Due to Cost Among Adults with Multimorbidity in the United States*, 12 Healthcare 2271, 2271-72, 2277, 2279 (2024). Azabuike and Alawode do an excellent job teasing out current research on the negative effect of price on accessing care for multiple categories of people, with a particular focus on the effect of comorbidities. *Id.* Given that we know people with less monetary resources do delay care due to price concerns, it would seem likely that excess pricing would both lead to higher rates of this behavior and excess profit leaving the system overall. A study of the effect of United States patents and drug pricing, comparing United States patents on medications and the access people have to those medicines, with other countries, gives some strong evidence that this problem does play out in this instinctively predictable manner, making tracking it seem to fit within the claims made here about excess pricing and access. For the drug patent pricing study, see Initiative for Medicines, Access, and Knowledge, Overpatented, Overpriced: A Data Brief on Medicare-Negotiated Drugs: Eliquis, Ozempic, Rybelsus and Wegovy 2-3, 5, 11 (2025), https://www.i-mak.org/overpatented/ [https://perma.cc/HJJ8-ZZZY].

10 For a detailed explanation of the role of price as a rationing mechanism in health care, see Jacqueline Fox, *Medicare Drug Price Negotiations: A Blueprint for Ethical Pricing Across the Healthcare System*, 19 FIU L. Rev. 71, 72, 91, 96, 103 (2025).

11
*See* David Merritt, *Unreasonable Markups by Corporate Hospital Systems Increase Prices*, BlueCross BlueShield (Oct. 9, 2024), https://www.bcbs.com/news-and-insights/article/unreasonable-markups-by-corporate-hospital-systems-increase-prices [https://perma.cc/ERE8-KWC7] (discussing increases in medical costs from Hospital Outpatient Department fees); Neil McCray, *Rent-Seeking in Medicaid Managed Care*, *in* Institutions and Incentives in Public Policy: An Analytical Assessment of Non-Market Decision-Making 81, 81-86 (Rosolino A. Candela et al. eds., 2022) (critiquing profit incentives stemming from fee-for-service and private managed care organization systems in Medicaid); Joseph Heath, *Ethical Issues in Physician Billing Under Fee-for-Service Plans*, 45 J. Med. & Phil. 86, 89-90 (2020) (describing the normalized professional culture of physician billing to increase billable value of services rendered).

12 For a simple discussion of funds subject to SEC reporting and the types of information disclosed, see *Fund Disclosure at a Glance*, U.S. Sec. & Exch. Comm’n (Oct. 23, 2024), https://www.sec.gov/about/divisions-offices/division-investment-management/fund-disclosure-glance [https://perma.cc/V2U2-MELN]. For a similar discussion as to how private equity investment funds are not subject to this requirement, see *Private Equity Funds*, U.S. Sec. & Exch. Comm’n, https://www.investor.gov/introduction-investing/investing-basics/investment-products/private-investment-funds/private-equity [https://perma.cc/J2HF-TDTL] (last visited June 24, 2025). For a detailed study of private equity differences from public investment funds, including the ramifications of SEC filing requirements, see generally Eileen Appelbaum & Rosemary Batt , *Private Equity Buyouts in Healthcare: Who Wins, Who Loses?* 7-8 (Inst. for New Econ. Thinking, Working Paper No. 118, 2020), 10.36687/inetwp118.

13
*See* Lawrence O. Gostin et al., *An Analysis of the Blizzard of Lawsuits to Block Drug-Price Negotiations*, MedPage Today (Aug. 9, 2023), https://www.medpagetoday.com/opinion/the-health-docket/105818?trw=no [https://perma.cc/TVB9-U4KF]. An example of this is the number of lawsuits filed by the drug industry in an effort to avoid participating in Medicare’s drug price negotiating process. *Id.* While one could argue this litigation is about avoiding price constraints, the focus of the process is forcing manufacturers to defend the reasonability of their pricing. For a discussion of this dynamic, see *Medicare Drug Price Negotiations: A Blueprint for Ethical Pricing Across the Healthcare System*, 19 FIU L. Rev. 71, 78 (2025). Were they comfortable with the imposition of that duty, i.e., if they felt they could readily show that their pricing allowed for a fair and reasonable return, they likely would not be as resistant.

14 Starting in the 1940s, Oskar Morgenstern wrote a series of books and article examining how problematic these types of numbers can be. *See* Oskar Morgenstern, *The Theory of Games*, 180 Sci. Am., no. 5, May 1949, at 22, 22-25 (analyzing game theory as it applies to social and economic behavior); Oskar Morgenstern, *Oligopoly, Monopolistic Competition, and the Theory of Games*, 38 Am. Econ. Rev., no. 2, May 1948, at 10, 10-18 (applying game theory to imperfect economic competition like oligopoly and monopoly). *See generally* John von Neumann & Oskar Morgenstern, Theory of Games and Economic Behavior (1944) (introducing game theory and gamesmanship); Oskar Morgenstern, On the Accuracy of Economic Observations (2d ed. 1965) (critiquing the reliance of data on national income as an indicator of economic health and policies). In an article in Fortune magazine summarizing his work, he explains that when collecting this type of economic data, there is often a “deliberate attempt to hide information.” Oskar Morgenstern, *Qui Numerare Incipit Errare Incipit* [*He Who Begins to Count Begins to Err*], Fortune Mag., Oct. 1963, at 143, 143.

15 See Fox, *supra* note 10 for a full discussion and citations to support this claim. *See also* Health Catalyst Eds., *Three Cost-Saving Strategies to Reduce Healthcare Spending*, HealthCatalyst, https://www.healthcatalyst.com/learn/insights/healthcare-cost-saving-strategies [https://perma.cc/WP5N-P93A] (last visited Sep. 29, 2025) (suggesting physician engagement, patient payment prediction models, and evidence-based standardization of care processes as mechanisms for healthcare cost reduction).

16 When polled, physicians express these concerns and sentiments, see, for example, Michael DePeau-Wilson, *Most Physicians Down on Private Equity in Healthcare*, MedPage Today (Mar. 11, 2024), https://www.medpagetoday.com/special-reports/features/109108 [https://perma.cc/K5MF-3TV5] ("The first [editor’s note for the study] concerns confronting the moral injury among clinicians who ’may be torn between two conflicting agendas: (1) the physical and mental health of their patients, and (2) the financial health of the health systems’ owners.’" (alterations in original)). It is common for data to show decreases in the quality of patient outcomes after private equity investments in practices or hospitals, see, for example, this bipartisan Senate report from January 2025, Staff of S. Budget Comm., 118th Cong., Report on Profits Over Patients: The Harmful Effects of Private Equity on the U.S. Health Care System ii-iv, 4, 7 (2025), https://www.budget.senate.gov/imo/media/doc/profits_over_patients_the_harmful_effects_of_private_equity_on_the_ushealthcaresystem1.pdf [https://perma.cc/N5P6-RD4H].

17 A 2023 large multinational meta-analysis of other studies tracking the impact of private equity investments on health care found that “[a]cross the outcome measures, PE ownership was most consistently associated with increases in costs to patients or payers. Additionally, PE ownership was associated with mixed to harmful impacts on quality.” Alexander Borsa et al., *Evaluating Trends in Private Equity Ownership and Impacts on Health Outcomes, Costs, and Quality: Systematic Review*, BMJ, June 11, 2023, at 1, 382 (alteration in original), https://doi.org/10.1136/bmj-2023-075244. Common methods for calculating health care spending such as the System of Health Accounts 2011 do not have any categories that identify this type of spending. *See* Org. for Econ. Coop. & Dev. et al., A System of Health Accounts 2011, at 183, 199 (rev. ed. 2017), https://www.oecd.org/content/dam/oecd/en/publications/reports/2017/03/a-system-of-health-accounts-2011_g1g75c9d/9789264270985-en.pdf [https://doi.org/10.1787/9789264270985-en]. *See* Sneha Kannan et al., *Changes in Hospital Adverse Events and Patient Outcomes Associated With Private Equity Acquisition*, 330 [J]AMA 2365, 2365-75 (2023) (looking at the interaction between private equity acquisition and the changes it imposes on hospital adverse events and patient outcomes).

18
*See* Bugbee, *infra* note 19. As discussed below in the case study, sale-leasebacks of underlying real estate is an example. *See infra* note 19 and accompanying text. The sale of the practice by senior members to private equity, which finances the purchase through debt held by the practice, is another method that results in debt payments that must be made by a practice or hospital; for an explanation of these purchase structures, see Andrew Schlafly, *The Harm from Private Equity’s Takeover of Medical Practices and Hospitals*, 121 Mo. Med. 328, 328-30 (2024).

19
*See, e.g.*, Mary Bugbee, Priv. Equity Stakeholder Project, The Pillaging of Steward Health Care: How a Private Equity Firm and Hospital Landlord Contributed to Steward’s Bankruptcy 3-22 (2024), https://pestakeholder.org/wp-content/uploads/2024/07/PESP_report_Steward-Bankruptcy_2024.pdf [https://perma.cc/A2DQ-EVDQ] (exemplifying MPT impacts on struggling hospitals in the United States).

20
*See* Maya Brownstein, *Private Equity’s Appetite for Hospitals May Put Patients at Risk*, Harv. T.H. Chan Sch. Pub. Health (Dec. 16, 2024), https://hsph.harvard.edu/news/private-equitys-appetite-for-hospitals-may-put-patients-at-risk/ [https://perma.cc/E687-77LF] (describing sale-leaseback agreement between MPT and Steward Health Care, a struggling Massachusetts hospital system which later filed for bankruptcy following inability to complete timely rent payments to MPT). Many states allow a nonprofit hospital that is exempt from property tax to pass that exemption through to the lessee, preserving that substantial benefit in these arrangements. For a comparison of state tax treatment of property owned by nonprofit organizations, see Joseph J. Cordes, *Assessing the Nonprofit Property Tax Exemption: Should Nonprofit Entities Be Taxed for Using Local Public Goods?*, *in* Value Capture and Land Policies 353, 355-64 (Gregory K. Ingram & Yu-Hong Hong eds., 2012).

21 Bugbee, *supra* note 19, at 6, 10, 17, 20.

22 Examples of this include Pipeline Health and Steward Healthcare System, both of which have these complex and debt-laden transaction histories with MPT; Steward is discussed in a case study. Bugbee, *supra* note 19, at 6, 10, 17, 20. See Maureen Tkacik, *Let Them Eat Invoices*, Am. Prospect (Aug. 13, 2024), https://prospect.org/health/2024-08-13-let-them-eat-invoices/ [https://perma.cc/RBA6-7S32] for examples of MPT’s newsworthy problems related to cash-strapped hospitals in California, Louisiana, and Alabama. In 2022, executives from MPT-affiliated companies used funding from MPT to found and then bankrupt Pipeline, a small hospital chain that remains behind on debts. *Id.* For an example of a sale leaseback agreement that predated bankruptcy protection, see *Pipeline Health Partners with Medical Properties Trust in Los Angeles*, Pipeline Health (July 29, 2021), https://www.pipelinehealth.us/newsroom/news/pipeline-health-partners-with-medical-properties-trust-in-los-angeles/ [https://perma.cc/4AER-VZYT]. MPT was also closely involved in financing Pipeline’s bankruptcy reorganization. *See, e.g.*, *Medical Properties Trust Announces That Pipeline Health Will Assume the Existing Terms of Its Los Angeles Hospital Master Lease*, BusinessWire (Jan. 13, 2023), https://www.businesswire.com/news/home/20230113005440/en/Medical-Properties-Trust-Announces-That-Pipeline-Health-Will-Assume-the-Existing-Terms-of-Its-Los-Angeles-Hospital-Master-Lease [https://perma.cc/97JZ-7AM2]. A different type of systematic wealth extraction can be seen when nonprofit hospital management firms acquire community hospitals with the intention of closing them to extract the value of the community hospital and divert funds to more lucrative medical institutions and services. *See* Zachary J. Gallin & Emily L. Xu, *Private Equity Strategies in Nonprofit Health Care*, 27 AMA J. Ethics 354, 355-56 (2025). When nonprofit health systems charge extraordinary prices to nonprofit hospitals, the owner of the hospital (often related to the management company) can then sell the hospital’s real estate for almost no value, due to how debt-laden and, ultimately, valueless, the hospital has become. For an example of this that was discovered only due to a lengthy government investigation, see the details surrounding the still-ongoing Providence Hospital/Ascension transactions over the last six years. *See* Press Release, Off. of the Att’y Gen. for D.C., Investigating the Closure of Providence Hospital & Safeguarding Nonprofit Assets (Sep. 8, 2020), https://oag.dc.gov/blog/investigating-closure-providence-hospital [https://perma.cc/2MUF-PYFJ] (finding that fees charged by nonprofit health care network on a large nonprofit hospital were excessive and intended to force sale of hospital real estate to recover debt incurred).

23 Bugbee, *supra* note 19, at 3, 8.

24
*Id.*

25
*Id.* at 3.

26
*Id.*

27
*Id.* at 3-4, 6, 10-12, 21.

28
*Id.* at 4.

29
*Id.* (alteration in original).

30
*See Examining the Bankruptcy of Steward Health Care: How Management Decisions Have Impacted Patient Care: Hearing Before the S. Comm. On Health, Educ., Lab., & Pensions*, 118th Cong. 10-14 (2024) [hereinafter *Hearings*] (statement of Audra Sprague, R.N., Former Nurse at Nashoba Valley Med. Ctr. Lunenburg, MA). *See also id.* at 6-10 (statement of Ellen MacInnis, R.N., Nurse at St. Elizabeth’s Med. Ctr., Boston, MA).

31
*See Hearings*, *supra* note 30, at 8-9 (statement of Ellen MacInnis, R.N.).

32 Tkacik, *supra* note 22.

33
*See Hearings*, *supra* note 30, at 9 (statement of Ellen MacInnis, R.N.).

34
*See Hearings*, *supra* note 30, at 13-14 (statement of Audra Sprague, R.N.); *id.* at 8-9 (statement of Ellen MacInnis, R.N.).

35
*See* Tkacik, *supra* note 22.

36 See Bobby Yu, Peng Liu & Prashant Das, *Investor Networks and Fund Performance in Private Equity Real Estate Funds*, J. Real Est. Fin. & Econ., Jan. 2025, at 1, 31, 39, for a study of investors in private equity real estate funds, for example, showed how, given that fund information is not readily available to the public, connections and relationships determined who participated in and profited from the funds.

37 See Brown & Hall, *supra* note 4, at 532-33, 543-45, for a discussion of these harms.

38 For example, Jeny Maier, Monica Smith & Leandra Ipina, Glob. Competition Rev., Americas Antitrust Review: Why US Regulators are Cracking Down on Private Equity Investments in the Healthcare Sector (2025); Daniel L. Fahey & Timothy J. Murphy, *2025 Picks Up Steam with Increased Scrutiny of Health Care Transactions and Corporate Structures*, Health L. Advisor, (Mar. 6, 2025), https://www.healthlawadvisor.com/2025-picks-up-steam-with-increased-scrutiny-of-health-care-transactions-and-corporate-structures [https://perma.cc/6EMF-HV9N].

39
*See* Schlafly, *supra* note 18, at 329-30 (discussing the wealth extraction practices that private equity groups utilize to make money off of health care without providing care). People who profit from different forms of financialization can also be health care providers; they are not mutually exclusive activities. *Id.* at 330-31. Selling to a private equity firm, for example, usually gives a physician access to a large amount of cash which is taxed at lower rate than income is. *See id.* (“The cash enticement can be very appealing to a physician. Private equity typically pays 15 times the physician’s annual income to acquire it entirely, or a smaller proportional amount to acquire as little as 30% of it. That purchase price may then be taxed at the lower capital gains rate, leaving the physician with a handsome payday and an opportunity to retire early.”). However, these activities ought to be analyzed differently and not blended together. Blending risks the same problems with masking that current accounting does, allowing providers, hospital administrators, and others in the field to hide excess profit within otherwise ethically acceptable work.

40 See generally Davis Tornabene, *Compendium of Codes of Ethics for Health Care Professionals—And Why They Matter*, Univ. Mia. Fla. Bioethics Network (Apr. 2025), https://fbn.miami.edu/resources/codes-of-ethics-for-health-professionals/index.html [https://perma.cc/4LGQ-H7HA] for a collection of ethical codes across different medical professions.

41 See Nicholas Bagley, *Medicine as a Public Calling*, 114 Mich. L. Rev. 57, 59 (2015), for discussion of how, in the context of the health care system as a public utility, it is commonly understood that the life or death quality of what is being bought and sold in health care distorts how consumers interact with the marketplace.

42 See Matt Zwolinski, Benjamin Ferguson & Alan Wertheimer, *Exploitation*, Stan. Encyclopedia Phil. (Dec. 20, 2001), https://plato.stanford.edu/entries/exploitation/ [https://perma.cc/BP5A-V7H2], for an explanation of how the term “exploitation” is used here as it is used in philosophy, whereby in its very definition, it is considered unethical.

43 The public utility discussion, *infra*, is an example of how to approach this problem by structuring a system that minimizes the risk.

44 See Fox, *supra* note 10, at 72, 103 for a detailed discussion of this issue.

45
*See* Livio Garattini & Anna Padula, *Competition in Health Markets: Is Something Rotten?*, 112 J. Royal Soc’y Med. 6, 8-9, (2019) (studying the usefulness of health care competition in Europe for the first roughly 20 years of this century). “Market competition in health care is not supported by economic theory and required ideological support from the very first British attempt in Europe, which very likely stemmed from a reform driven for political purposes against purely public systems, generating — perhaps inevitably — muddle in the long run.” *Id.* at 8.

46
*See* Borsa et al., *supra* note 17, at 13 (discussing changes to staffing towards less expensive non-physician clinicians).

47 See Nita Khandelwal et al., *The Patient and Family Member Experience of Financial Stress Related to Critical Illness*, 23 J. Palliative Med. 972, 974-75 (2020), for a study of patient stress from financial concerns; see also Sammer Marzouk, Lucy Tu, & Fatima C. Stanford, *Financial Crossroads of Care: Physicians’ Struggle and Patient Outcomes*, 30 Am. J. Managed Care 305, 305-07 (2024), for a discussion of how reimbursement rate reductions can contribute to physician burn out.

48 For an example webpage promoting the benefits of private equity for patients, see *Hospitals Depend on Private Equity to Support Better Patient Outcomes and Improve Efficiencies*, Am. Inv. Council (Apr. 15, 2024), https://www.investmentcouncil.org/hospitals-depend-on-private-equity-to-support-better-patient-outcomes-and-improve-efficiencies/ [https://perma.cc/4NQ8-3SKJ]. Interestingly, the studies it cites in defense of its claims are almost all from business schools and consulting firms. *See id.*

49 DePeau-Wilson, *supra* note 16; Staff of S. Budget Comm., *supra* note 16, at iv-v; Borsa et al., *supra* note 17, at 1, 13; Kannan et al., *supra* note 17, at 2371, 2373; Bugbee, *supra* note 19, at 18-19, 23; *Hearings*, *supra* note 30, at 8-9, 13-14; Brown & Hall, *supra* note 4, at 532-33, 548; Marzouk, Tu & Stanford, *supra* note 47, at 306.

50 For an example on how these deals are complex and subject to intense criticism, see Sharon Reynolds, *Infections and Falls Increased in Private Equity-Owned Hospitals*, Nat’l Insts. of Health, (Jan. 23, 2024), https://www.nih.gov/news-events/nih-research-matters/infections-falls-increased-private-equity-owned-hospitals [https://perma.cc/GNY8-3P2J]. The negative effect on health care is extremely well documented. *See id.*; sources cited *supra* note 49.

51 Staff of S. Budget Comm., *supra* note 16, at 46-47; Bugbee, *supra* note 19, at 16, 20.

52 Staff of S. Budget Comm., *supra* note 16, at 5, 48; Bugbee, *supra* note 19, at 23-24, 30, 32.

53 Note the phrase “ethical framing” is used intentionally here. This is not the same as a method that can concretely determine prices, but rather an acknowledgment that pricing has ethical implications and ought to be ethically defensible.

54 For an example why we often use communal resources to fund water systems, see James McBride & Noah Berman, *How U.S. Water Infrastructure Works*, Council on Foreign Rels. (May 2, 2024), https://www.cfr.org/backgrounder/how-us-water-infrastructure-works [https://perma.cc/HQU3-AVYK]. This allows for spreading the cost across those who benefit and avoiding the risk of having water held hostage for someone’s profit.

55
*See* Zwolinski, Ferguson & Wertheimer, *supra* note 42. It is commonly understood in moral philosophy that exploitation, properly defined, is ethically wrong. *Id.* The Stanford Encyclopedia of Philosophy, in its article on exploitation, puts it this way: To exploit someone is to take unfair advantage of them. It is to use another person’s vulnerability for one’s own benefit. Of course, benefitting from another’s vulnerability is not always morally wrong—we do not condemn a chess player for exploiting a weakness in his opponent’s defence, for instance. But some forms of advantage-taking do seem to be clearly wrong, and it is this *normative* sense of exploitation that is of primary interest to moral and political philosophers.
*Id.*

56 For an example of this scenario referred to as a “‘hostage’ bargaining model” in drug pricing, see Frederick M. Abbot, *Excessive Pharmaceutical Prices and Competition Law: Doctrinal Development to Protect Public Health*, 6 U.C. Irvine L. Rev. 281, 303-04 (2016).

57
*See* Bagley, *supra* note 41, at 59 (alteration in original).

58
*See generally* Govind Persad, *Pricing Drugs Fairly*, 62 Wm. & Mary L. Rev. 929 (2021) (arguing for a notion of fairness that derives from the social value of the drug, and a price ceiling that is tied to that social value).

59
*Id.*

60 As an example of this dynamic, see Sydney Lupkin, *Health Insurers Cover Fewer Drugs and Make Them Harder to Get*, NPR: Health News (June 27, 2024, at 16:18 ET), https://www.npr.org/2024/06/27/g-s1-6773/health-insurers-cover-fewer-drugs-and-make-them-harder-to-get [https://perma.cc/G8D6-8ALU]. NPR did a story covering negotiations between drug companies and insurance companies, where drug companies refuse to lower their prices, so the insurance companies do not cover that specific medicine. *Id.*

61 For a discussion of the current Federal efforts to negotiate drug prices, see Fox, *supra* note 10, at 74-75.

62
*See* Michael E. Porter, *What Is Value in Health Care?*, 363 New Eng. J. Med. 2477, 477 (2010).

63 Porter, *supra* note 62, at 2477.

64
*Id.*

65 Jesse Welles, *The Poor*, *on* The Poor [Single], at 00:09-00:19 (July 26, 2024).

66
*Id.*

67 Hardaway and Mcloyd have a highly cited article from 2008 collecting studies showing how societal structures make it difficult to use work as a reliable method to leave poverty. *See generally* Cecily R. Hardaway & Vonnie C. Mcloyd, *Escaping Poverty and Securing Middle Class Status: How Race and Socioeconomic Status Shape Mobility Prospects for African Americans During the Transition to Adulthood*, 38 J. Youth & Adolescence 242 (2014) (demonstrating how work cannot be relied on as a method to leave poverty).

68 For discussion and early research on this topic, including contradicting data, see David Orentlicher, *Controlling Health Care Spending: More Patient “Skin in the Game?”*, William S. Boyd Sch. of L. Scholarly Works, 2016, at 348, 349-54; and see generally Joel S. Weissman et al., *Delayed Access to Health Care: Risk Factors, Reasons, and Consequences*, 114 Annals Internal Med. 325 (1991) (arguing there is little to no data showing patterns of overuse of medical care that is driven by patients, but there is much data showing the opposite, that a significant percentage of patients persistently delay care when it is necessary if patients fear they cannot afford it).

69 For a discussion of this in further detail and accompanying citations, see Jacqueline R. Fox, *The Lived Experience of Health Insurance: An Analysis and Proposal for Reform*, 14 Ne. U.L. Rev. 429, 452-57 (2022).

70
*Id.*

71
*See* Jing Fang et al., *Awareness of Heart Attack Symptoms and Response Among Adults — United States, 2008, 2014, and 2017*, Ctrs. for Disease Control & Prevention (Feb. 8, 2019), https://www.cdc.gov/mmwr/volumes/68/wr/mm6805a2.htm [https://perma.cc/A4SV-UKPD]; *Signs and Symptoms of a Stroke*, Ctrs. for Disease Control & Prevention (Oct. 24, 2024), https://www.cdc.gov/stroke/signs-symptoms/index.html [https://perma.cc/DK2E-C7DV].

72
*See* Fox, *supra* note 69, at 452-53. For example, the size of unmet deductibles and ability to pay them lead to dramatic differences in how long it takes people to seek care for a heart attack that is actually occurring see generally *id.* (discussing the impact of unmet deductibles and ability to pay on medical decision making).

73
*See* Elina Dale et al., *Criteria for the Procedural Fairness of Health Financing Decisions: A Scoping Review*, 38 Health Pol’y & Plan. i13, i25-i26 (2023).

74
*See* Munira Z. Gunja, Evan D. Gumas & Reginald D. Williams II, *U.S. Health Care from a Global Perspective, 2022: Accelerating Spending, Worsening Outcomes*, The Commonwealth Fund (Jan. 31, 2023), https://www.commonwealthfund.org/publications/issue-briefs/2023/jan/us-health-care-global-perspective-2022 [https://doi.org/10.26099/8ejy-yc74].

75
*Id.*

76
*See America’s Health Rankings: International Comparison*, United Health Found. (2023), https://www.americashealthrankings.org/learn/reports/2023-annual-report/international-comparison [https://perma.cc/NN9P-CJPA].

77
*Id.*; Munira Z. Gunja et al., *Insights into the U.S. Maternal Mortality Crisis: An International Comparison*, The Commonwealth Fund (June 4, 2024), https://www.commonwealthfund.org/publications/issue-briefs/2024/jun/insights-us-maternal-mortality-crisis-international-comparison [https://doi.org/10.26099/cthn-st75].

78 OECD, Health at a Glance 2025: OECD Indicators 68-69, https://doi.org/10.1787/8f9e3f98-en; *see* David Blumenthal et al., *Mirror, Mirror 2024: A Portrait of the Failing U.S. Health System*, The Commonwealth Fund (Sep. 19, 2024), https://www.commonwealthfund.org/publications/fund-reports/2024/sep/mirror-mirror-2024 [https://doi.org/10.26099/ta0g-zp66].

79
*See* Caroline Barakat & Theodore Konstantinidis, *A Review of the Relationship Between Socioeconomic Status Change and Health*, 20 Int’l J. Env’t Rsch. & Pub. Health 6249, 6249 (2023).

80
*See* Wendy L. Macias-Konstantopoulos et al., *Race, Healthcare, and Health Disparities: A Critical Review and Recommendations for Advancing Health Equity*, 24 W.J. Emergency Med. 906, 907-11 (2023).

81 Kenzie A. Cameron et al., *Gender Disparities in Health and Healthcare Use Among Older Adults*, 19 J. Women’s Health 1643, 1648-49 (2010).

82 Dhruv Kullar & Dave A. Chokshi, *Health, Income, & Poverty: Where We Are & What Could Help*, Health Affs.: Health Pol’y Brief (Oct. 4, 2018).

83
*Healthy People 2030: Enrollment in Higher Education*, U.S. Dep’t Health & Hum. Servs., https://odphp.health.gov/healthypeople/priority-areas/social-determinants-health/literature-summaries/enrollment-higher-education#:~:text=College%20graduates%20have%20better%20self,12 [https://perma.cc/R7CH-ZHW2] (last visited Sep. 4, 2025).

84
*See* John B. Dixon, *The Effect of Obesity on Health Outcomes*, 316 Molecular & Cellular Endocrinology 104, 104-07 (2010).

85
*See generally* Tatyana Deryugina & David Molitor, *The Causal Effects of Place on Health and Longevity*, 35 J. Econ. Perspectives 147 (2023) (explaining the impacts of geography and environmental stressors on health outcomes in U.S. regions and internationally).

86
*See* Amelia Whitman et al., Dep’t Health & Hum. Servs.: Off. Health Pol’y, Addressing Social Determinants of Health: Examples of Successful Evidence-Based Strategies and Current Federal Efforts 1-2 (2022).

87
*See* Andy Davis et al., *US Health Care Can’t Afford Health Inequities*, Deloitte Insights (June 22, 2022), https://www.deloitte.com/us/en/insights/industry/health-care/economic-cost-of-health-disparities.html [https://perma.cc/9NSG-K9U9].

88
*See, e.g.*, Joni J. Young, *Constructing, Persuading, and Silencing: The Rhetoric of Accounting Standards*, 28 Acct., Orgs. & Soc’y 621, 637-38 (2003). Within accounting, it is generally understood that, at least arguably, accounting standards and the methodology for creating accounting standards have a rhetorical weight that implies accounting results have a higher degree of accuracy than they can reasonably be expected to have. *Id.* at 637. This use of rhetorical communication has been described as doing the “work to maintain the myth of accounting objectivity.” *Id.*

89
*See id.* at 621-22 (discussing the standard setting process and the impact that it can have on resulting numbers).

90
*See id.* at 621, 625-26.

91
*See id.* at 637.

92 Health Expenditures Highlights, *supra* note 3.

93
*See, e.g.*, Young, *supra* note 88, at 637 (discussing the importance of accounting standards such that the standards board acts as a gatekeeper).

94
*See generally id.* (exploring the discussions and factors that are considered in setting accounting standards).

95
*See, e.g.*, Emma Wager, Matt McGough, Shameek Rakshit & Cynthia Cox, *How Does Health Spending in the U.S. Compare to Other Countries?*, Peterson-KFF: Health Sys. Tracker (Apr. 9, 2025), https://www.healthsystemtracker.org/chart-collection/health-spending-u-s-compare-countries/ [ https://perma.cc/LR8A-8DUQ].

96
*See Why Are Americans Paying More for Healthcare?*, Peter G. Peterson Found. (Aug. 12, 2025), https://www.pgpf.org/article/why-are-americans-paying-more-for-healthcare/ [https://perma.cc/LKK4-FAR2] (discussing U.S. per capita health care spending in comparison to other countries).

97
*Id.* (reporting that despite higher spending, the U.S. does not have better health outcomes).

98 Daniel Mügge, *Economic Statistics as Political Artefacts*, 29 Rev. Int’l Pol. Econ., no. 1, 2022, at 1, 3.

99 The seminal author in this area is probably Oskar Morgenstern, whose 1950 book (updated through the 1960s), On the Accuracy of Economic Observations (2d ed. 1963), and subsequent publications provided a necessary counterweight to perceptions of technical perfection in economic statistics. *See also* Oskar Morgenstern, *Thirteen Critical Points in Contemporary Economics Theory: An Interpretation*, 10 J. Econ. Literature 1163 (1972).

100
*See* Mügge, *supra* note 98, at 8-9.

101
*Id.*

102
*Id.*

103
*See, e.g.*, *Healthcare Expenditure, UK Health Accounts: 2023 and 2024*, Off. for Nat’l Stat. (Apr. 30, 2025), https://www.ons.gov.uk/peoplepopulationandcommunity/healthandsocialcare/healthcaresystem/bulletins/ukhealthaccounts/2023and2024 [https://perma.cc/A6DN-MWS5]; *National Health Expenditure Data: Historical*, Ctrs. for Medicare & Medicaid Servs. (Dec. 18, 2024, at 16:28 ET), https://www.cms.gov/data-research/statistics-trends-and-reports/national-health-expenditure-data/historical [https://perma.cc/C7VV-TVAP].

104
*National Health Expenditure Data: Historical*, *supra* note 103.

105
*Id.*

106
*Id.*

107
*Id.* The United Kingdom is slightly more cautious than the United States when it presents these numbers. *See Healthcare Expenditure, UK Health Accounts: 2023 and 2024*, *supra* note 103.

108 Christopher Cai et al., *Projected Costs of Single-Payer Healthcare Financing in the United States: A Systematic Review of Economic Analyses*, 17 PLOS Med., Jan. 15, 2020, at 1, 14, https://journals.plos.org/plosmedicine/article/file?id=10.1371/journal.pmed.1003013&type=printable [https://doi.org/10.1371/journal.pmed.1003013].

109
*Id.* at 7, 12.

110
*See A Single-Payer Health Care System That Is Based on Medicare’s Fee-for-Service Program: Hearing Before the S. Comm. on the Budget*, 117th Cong. 1 (2022) (testimony document of Philip L. Swagel, Director, Cong. Budget Off.).

111
*See Trends in Health Care Spending*, AMA (Apr. 17, 2025), https://www.ama-assn.org/about/ama-research/trends-health-care-spending [https://perma.cc/G6X8-QUCQ] (breaking down costs and omitting behavioral costs).

112
*See* Cathy Nelson, *The Correlation Between Overutilization of Services & Lost Revenue*, MCG Health (Apr. 3, 2024), https://www.mcg.com/blog/overutilization-lost-revenue-consulting/ [https://perma.cc/RK44-CQK5]; Daniel Crespin et al., *Upcoding Linked to Up to Two-Thirds of Growth in Highest-Intensity Hospital Discharges in 5 States, 2011-19*, 43 Health Affs. 1619, 1624-25 (2024); Beth Duff-Brown, *When Healthcare Providers Go Bankrupt, Patients Pay the Price*, Stan. Health Pol’y (May 12, 2025), https://healthpolicy.fsi.stanford.edu/news/when-healthcare-providers-go-bankrupt-patients-pay-price [https://perma.cc/3NPY-B6FA].

113
*See* Roosa Tikkanen et al., *International Health Care System Profiles: England*, The Commonwealth Fund (June 5, 2020), https://www.commonwealthfund.org/international-health-policy-center/countries/england [https://perma.cc/7HGH-JGVK]; David Rowland, *Investors Are Making a Fortune from UK Healthcare. Why Is Nobody Holding Private Equity to Account?*, The Guardian (Mar. 13, 2024), https://www.theguardian.com/commentisfree/2024/mar/13/uk-healthcare-private-equity-cancer-treatment-services [https://perma.cc/FM99-WMGD].

114 Rowland, *supra* note 113.

115
*See* Hettie O’Brien, *Private Equity Has Its Sights on the NHS–and With it Our Faith in Public Services Altogether*, The Guardian (Aug. 17, 2023), https://www.theguardian.com/commentisfree/2023/aug/17/private-equity-nhs-hospital-crisis [https://perma.cc/2X5Q-8HXP] (collecting politicians’ comments and data on problematic impacts on patient outcomes and cost).

116
*See* Denis Campbell, *Private Sector’s Role in Cutting NHS Waiting Lists in England to Rise by 20%*, The Guardian (Jan. 6, 2025), https://www.theguardian.com/politics/2025/jan/06/private-sector-cutting-nhs-waiting-lists-england-keir-starmer [https://perma.cc/XK7B-TTRU].

117
*See* NHS Support Fed’n, *Private Equity —“Decimated” US Hospitals According to Report—Now Targeting the NHS*, The Lowdown (Sep. 2, 2025), https://lowdownnhs.info/private-providers/private-equity-destroyer-of-us-healthcare-now-targeting-the-nhs/ [https://perma.cc/ZDD2-4CQH] (discussing the attraction of private equity to healthcare markets); Rowland, *supra* note 113 (discussing the potential for returns for companies backed by private equity funds).

118 Laura Webster, *Wes Streeting Admits Labour Would Use Private Firms to Tackle NHS Waiting Lists*, The Nat’l (Jan. 8, 2022), https://www.thenational.scot/news/19833928.wes-streeting-admits-labour-use-private-firms-tackle-nhs-waiting-lists/ [https://perma.cc/362Q-JVWS].

119 In the United States, this would be the amount calculated using the annual national health care expenditures described earlier. *See National Health Expenditure Data: Historical*, *supra* note 103.

120 All of these calculations are subject to limitations on their correctness. Making the claim that these numbers show us any amounts with surety is, by itself, an exercise in the rhetoric of accounting. A full discussion of all such limitations is outside the scope of this article.

121 Peter Whoriskey, *Private Equity Investors Raising Prices U.S. Medical Prices, Study Says*, Wash. Post (July 10, 2023, at 12:08 ET), https://www.washingtonpost.com/business/2023/07/10/private-equity-raising-prices-doctors-practices-private-equity-doctors/ [https://perma.cc/X6MW-WBKJ].

122 United States v. Carroll Towing Co., 159 F.2d 169, 173 (2d Cir. 1947).

123
*See id.* The result of these calculations is further constrained by a proximate cause analysis that protects some degree of both individual and societal benefit from internalizing the costs of the underlying, risky undertaking. *See id.* at 174.

124
*See id.* at 171-72.

